# Benzyl 2-{[2,8-bis­(trifluoro­meth­yl)quinolin-4-yl](hy­droxy)meth­yl}piperidine-1-carboxyl­ate

**DOI:** 10.1107/S1600536811047738

**Published:** 2011-11-16

**Authors:** Marcus V. N. de Souza, Raoni S. B. Gonçalves, James L. Wardell, Solange M. S. V. Wardell, Edward R. T. Tiekink

**Affiliations:** a Fundaçaõ Oswaldo Cruz, Instituto de Tecnologia, em Fármacos–Farmanguinhos, R. Sizenando Nabuco, 100, Manguinhos, 21041-250 Rio de Janeiro, RJ, Brazil; b Centro de Desenvolvimento Tecnológico em Saúde (CDTS), Fundação Oswaldo Cruz (FIOCRUZ), Casa Amarela, Campus de Manguinhos, Av. Brasil 4365, 21040-900 Rio de Janeiro, RJ, Brazil; c CHEMSOL, 1 Harcourt Road, Aberdeen AB15 5NY, Scotland; d Department of Chemistry, University of Malaya, 50603 Kuala Lumpur, Malaysia

## Abstract

The title mol­ecule, C_25_H_22_F_6_N_2_O_3_, adopts an open conformation whereby the quinoline and carboxyl­ate ester groups are orientated in opposite directions but to the same side of the piperidine ring so that the mol­ecule has an approximate U-shape. The piperidine ring adopts a distorted boat conformation. In the crystal, inversion dimers linked by pairs of O—H⋯O hydrogen bonds generate *R*_2_^2^(14) loops.

## Related literature

For background to the anti-mycobacterial activity of mefloquine, see: Gonçalves *et al.* (2010 ▶); Mao *et al.* (2007 ▶); Maguire *et al.* (2006 ▶). For the synthesis, see: Grellepois *et al.* (2005 ▶). For related structures, see: Gonçalves *et al.* (2011*a* ▶,*b* ▶); Wardell *et al.* (2010 ▶, 2011*a* ▶,*b* ▶); Pitaluga *et al.* (2010 ▶). For ring conformations, see: Cremer & Pople (1975 ▶).
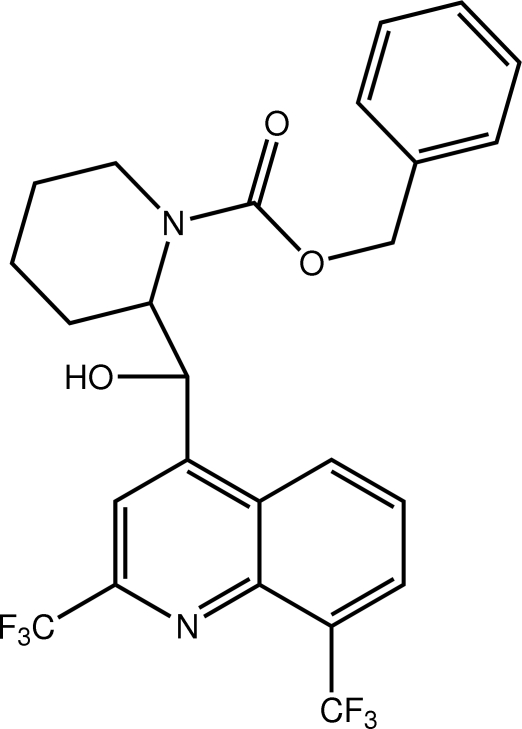


## Experimental

### 

#### Crystal data

C_25_H_22_F_6_N_2_O_3_
*M* *_r_* = 512.45
Monoclinic, 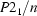

*a* = 12.7793 (5) Å
*b* = 13.9970 (7) Å
*c* = 13.2188 (9) Å
β = 109.999 (8)°
*V* = 2221.9 (2) Å^3^
*Z* = 4
Mo *K*α radiation
μ = 0.13 mm^−1^
*T* = 100 K
0.15 × 0.11 × 0.04 mm

#### Data collection

Rigaku Saturn724+ diffractometer
Absorption correction: multi-scan (*CrystalClear-SM Expert*; Rigaku, 2011 ▶) *T*_min_ = 0.757, *T*_max_ = 1.000
10271 measured reflections
5060 independent reflections
4132 reflections with *I* > 2σ(*I*)
*R*_int_ = 0.026

#### Refinement

*R*[*F* ^2^ > 2σ(*F* ^2^)] = 0.039
*wR*(*F* ^2^) = 0.107
*S* = 1.00
5060 reflections
328 parameters
1 restraint
H atoms treated by a mixture of independent and constrained refinement
Δρ_max_ = 0.34 e Å^−3^
Δρ_min_ = −0.32 e Å^−3^

### 

Data collection: *CrystalClear-SM Expert* (Rigaku, 2011 ▶); cell refinement: *CrystalClear-SM Expert*; data reduction: *CrystalClear-SM Expert*; program(s) used to solve structure: *SHELXS97* (Sheldrick, 2008 ▶); program(s) used to refine structure: *SHELXL97* (Sheldrick, 2008 ▶); molecular graphics: *ORTEP-3* (Farrugia, 1997 ▶) and *DIAMOND* (Brandenburg, 2006 ▶); software used to prepare material for publication: *publCIF* (Westrip, 2010 ▶).

## Supplementary Material

Crystal structure: contains datablock(s) global, I. DOI: 10.1107/S1600536811047738/hb6498sup1.cif

Structure factors: contains datablock(s) I. DOI: 10.1107/S1600536811047738/hb6498Isup2.hkl

Supplementary material file. DOI: 10.1107/S1600536811047738/hb6498Isup3.cml

Additional supplementary materials:  crystallographic information; 3D view; checkCIF report

## Figures and Tables

**Table 1:** Hydrogen-bond geometry (Å, °)

*D*—H⋯*A*	*D*—H	H⋯*A*	*D*⋯*A*	*D*—H⋯*A*
O1—H1*o*⋯O3^i^	0.84 (1)	1.90 (1)	2.7294 (14)	172 (2)

Symmetry code: (i) 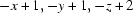.

**Fig. 1.**
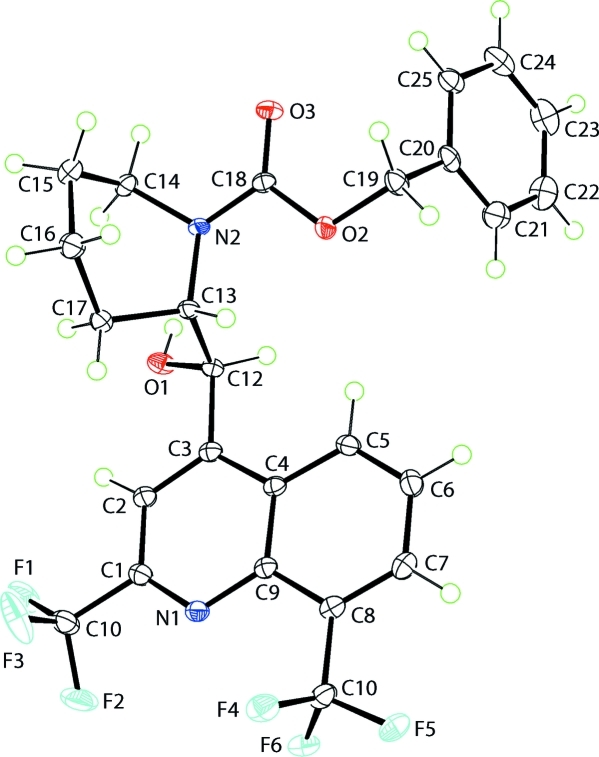
The molecular structure of (I) showing displacement ellipsoids at the 50% probability level.

**Fig. 2.**
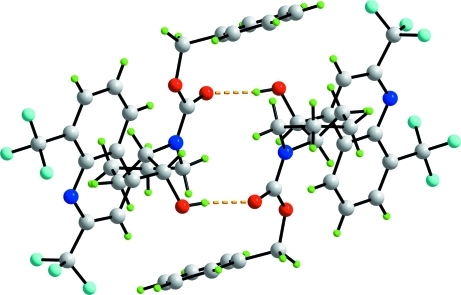
Centrosymmetric aggregate mediated by O—H···O hydrogen bonds (orange dashed lines) in the crystal structure of (I).

## supplementary crystallographic information

### Comment

For some decades, in combination with other drugs, mefloquine has been used in the prevention and treatment of malaria (Maguire *et al.*, 2006). The activity of mefloquine has been investigated against other diseases more recently, for example, as anti-viral and anti-tubercular agents (Mao *et al.*, 2007). In continuation of on-going structural and biological studies on mefloquine derivatives (Gonçalves *et al.*, 2010, 2011*a*, 2011*b*; Wardell, *et al.*, 2010; 2011*a*; 2011*b*; Pitaluga *et al.*, 2010), we now report the crystal and molecular structure of the title compound, (I).

In the molecule of (I), Fig. 1, the hydroxyl group lies to one side of the plane through the quinolinyl residue and the substituted piperidine ring to other with the carboxylate ester directed away from the rest of the molecule. The residues lie to the same side of the piperidine ring so that the molecule has a U-shape. The piperidine ring has a distorted boat conformation with ring puckering parameters: q_2_ = 0.7644 (16) Å; q_3_ = -0.0283 (16) Å; QT = 0.7649 (16) Å; and θ = 92.12 (12) ° (Cremer & Pople, 1975). Mefloquine used as a reagent was a racemate. The sum of the angles at the trisubstituted N2 is 356° indicating a very near planar geometry, and hence an achiral centre. The configurations of the C12 and C13 in the illustrated molecule, Fig. 1, are *R*, *S* and *R*, respectively. The crystal structure contains an equal amount of the opposite enantiomer.

The most prominent intermolecular interactions in the crystal structure are O—H···O hydrogen bonds that lead to the formation of centrosymmetric dimeric aggregates *via* 14-membered {···HOC_2_NCO}_2_ synthons, Fig. 1 and Table 1.

### Experimental

Benzyl 2-[[2,8-bis(trifluoromethyl)-4-quinolinyl](hydroxy)methyl]tetrahydro-1(2*H*)-pyridine carboxylate was prepared similarly to *tert*-butyl 2-[[2,8-bis(trifluoromethyl)-4-quinolinyl](hydroxy)methyl]tetrahydro-1(2*H*)-pyridine carboxylate, following a published procedure (Grellepois *et al.*, 2005), from benzyl chloroformate and mefloquine in the presence of Et_3_N. Colourless plates of (I) were grown from an EtOH solution; *M*.pt. 445–447 K. MS 535.3 [*M* + Na].

### Refinement

The C-bound H atoms were geometrically placed (C—H = 0.95–1.00 Å) and refined as riding with *U_iso_*(H) = 1.2*U_eq_*(C). The O—H H atom was located in a difference map and refined with O—H = 0.84±0.01 Å with *U_iso_*(H) = 1.5*U_eq_*(O).

### Crystal data

**Table:**

C_25_H_22_F_6_N_2_O_3_	*F*(000) = 1056
*M**_r_* = 512.45	*D*_x_ = 1.532 Mg m^−^^3^
Monoclinic, *P*2_1_/*n*	Mo *K*α radiation, λ = 0.71073 Å
Hall symbol: -P 2yn	Cell parameters from 8584 reflections
*a* = 12.7793 (5) Å	θ = 3.1–27.5°
*b* = 13.9970 (7) Å	µ = 0.13 mm^−^^1^
*c* = 13.2188 (9) Å	*T* = 100 K
β = 109.999 (8)°	Plate, colourless
*V* = 2221.9 (2) Å^3^	0.15 × 0.11 × 0.04 mm
*Z* = 4	

### Data collection

**Table:**

Rigaku Saturn724+ diffractometer	5060 independent reflections
Radiation source: Rotating Anode	4132 reflections with *I* > 2σ(*I*)
Confocal	*R*_int_ = 0.026
Detector resolution: 28.5714 pixels mm^-1^	θ_max_ = 27.5°, θ_min_ = 3.1°
profile data from ω–scans	*h* = −15→16
Absorption correction: multi-scan (*CrystalClear-SM Expert*; Rigaku, 2011)	*k* = −15→18
*T*_min_ = 0.757, *T*_max_ = 1.000	*l* = −14→17
10271 measured reflections	

### Refinement

**Table:**

Refinement on *F*^2^	Primary atom site location: structure-invariant direct methods
Least-squares matrix: full	Secondary atom site location: difference Fourier map
*R*[*F*^2^ > 2σ(*F*^2^)] = 0.039	Hydrogen site location: inferred from neighbouring sites
*wR*(*F*^2^) = 0.107	H atoms treated by a mixture of independent and constrained refinement
*S* = 1.00	*w* = 1/[σ^2^(*F*_o_^2^) + (0.0549*P*)^2^ + 0.9076*P*] where *P* = (*F*_o_^2^ + 2*F*_c_^2^)/3
5060 reflections	(Δ/σ)_max_ < 0.001
328 parameters	Δρ_max_ = 0.34 e Å^−^^3^
1 restraint	Δρ_min_ = −0.32 e Å^−^^3^

### Special details

**Table:**

Geometry. All s.u.'s (except the s.u. in the dihedral angle between two l.s. planes) are estimated using the full covariance matrix. The cell s.u.'s are taken into account individually in the estimation of s.u.'s in distances, angles and torsion angles; correlations between s.u.'s in cell parameters are only used when they are defined by crystal symmetry. An approximate (isotropic) treatment of cell s.u.'s is used for estimating s.u.'s involving l.s. planes.
Refinement. Refinement of *F*^2^ against ALL reflections. The weighted *R*-factor *wR* and goodness of fit *S* are based on *F*^2^, conventional *R*-factors *R* are based on *F*, with *F* set to zero for negative *F*^2^. The threshold expression of *F*^2^ > 2σ(*F*^2^) is used only for calculating *R*-factors(gt) *etc*. and is not relevant to the choice of reflections for refinement. *R*-factors based on *F*^2^ are statistically about twice as large as those based on *F*, and *R*- factors based on ALL data will be even larger.

### Fractional atomic coordinates and isotropic or equivalent isotropic displacement parameters (Å^2^)

**Table:**

	*x*	*y*	*z*	*U*_iso_*/*U*_eq_	
F1	0.06301 (8)	0.63866 (8)	0.42728 (8)	0.0347 (3)	
F2	0.08200 (10)	0.59309 (10)	0.27995 (8)	0.0472 (3)	
F3	0.00983 (8)	0.49890 (9)	0.36550 (11)	0.0490 (3)	
F4	0.24269 (7)	0.32103 (7)	0.22775 (7)	0.0244 (2)	
F5	0.40411 (8)	0.31616 (7)	0.21097 (7)	0.0268 (2)	
F6	0.32339 (7)	0.45128 (7)	0.20711 (7)	0.0223 (2)	
O1	0.34838 (8)	0.58357 (7)	0.75929 (8)	0.0177 (2)	
H1o	0.3897 (13)	0.6035 (13)	0.8197 (10)	0.026*	
O2	0.54803 (8)	0.32514 (8)	0.88013 (8)	0.0172 (2)	
O3	0.53313 (8)	0.33958 (7)	1.04525 (8)	0.0167 (2)	
N1	0.24628 (9)	0.47056 (9)	0.38278 (9)	0.0148 (2)	
N2	0.40608 (9)	0.40943 (8)	0.89798 (9)	0.0132 (2)	
C1	0.20188 (11)	0.52274 (10)	0.44060 (11)	0.0157 (3)	
C2	0.24724 (11)	0.53657 (10)	0.55218 (11)	0.0153 (3)	
H2	0.2115	0.5773	0.5879	0.018*	
C3	0.34448 (11)	0.49011 (10)	0.60906 (11)	0.0135 (3)	
C4	0.39716 (11)	0.43306 (10)	0.55111 (11)	0.0131 (3)	
C5	0.49874 (11)	0.38293 (10)	0.60028 (11)	0.0156 (3)	
H5	0.5336	0.3848	0.6763	0.019*	
C6	0.54664 (12)	0.33218 (11)	0.53960 (12)	0.0184 (3)	
H6	0.6149	0.2997	0.5737	0.022*	
C7	0.49589 (12)	0.32732 (10)	0.42648 (12)	0.0178 (3)	
H7	0.5305	0.2920	0.3852	0.021*	
C8	0.39727 (11)	0.37307 (10)	0.37610 (11)	0.0148 (3)	
C9	0.34484 (11)	0.42711 (10)	0.43713 (11)	0.0132 (3)	
C10	0.08955 (13)	0.56455 (12)	0.37809 (12)	0.0234 (3)	
C11	0.34203 (12)	0.36577 (11)	0.25637 (12)	0.0188 (3)	
C12	0.38962 (11)	0.49779 (10)	0.73029 (11)	0.0136 (3)	
H12	0.4728	0.5004	0.7553	0.016*	
C13	0.35295 (11)	0.40908 (10)	0.77995 (11)	0.0130 (3)	
H13	0.3785	0.3507	0.7515	0.016*	
C14	0.33521 (11)	0.43906 (11)	0.95997 (11)	0.0162 (3)	
H14A	0.3039	0.5032	0.9361	0.019*	
H14B	0.3805	0.4427	1.0374	0.019*	
C15	0.24059 (12)	0.36724 (12)	0.94326 (12)	0.0207 (3)	
H15A	0.1786	0.3981	0.9598	0.025*	
H15B	0.2674	0.3127	0.9931	0.025*	
C16	0.19855 (12)	0.33083 (11)	0.82632 (12)	0.0200 (3)	
H16A	0.2337	0.2685	0.8226	0.024*	
H16B	0.1169	0.3212	0.8024	0.024*	
C17	0.22634 (11)	0.40230 (10)	0.75142 (12)	0.0159 (3)	
H17A	0.1959	0.4659	0.7589	0.019*	
H17B	0.1916	0.3814	0.6757	0.019*	
C18	0.49755 (11)	0.35697 (10)	0.94887 (11)	0.0132 (3)	
C19	0.63991 (11)	0.25860 (11)	0.92685 (12)	0.0184 (3)	
H19A	0.6503	0.2196	0.8686	0.022*	
H19B	0.6203	0.2148	0.9764	0.022*	
C20	0.74791 (12)	0.30819 (10)	0.98754 (12)	0.0171 (3)	
C21	0.80837 (13)	0.35436 (11)	0.93193 (13)	0.0226 (3)	
H21	0.7803	0.3564	0.8555	0.027*	
C22	0.90963 (13)	0.39735 (12)	0.98804 (15)	0.0272 (4)	
H22	0.9506	0.4285	0.9498	0.033*	
C23	0.95114 (13)	0.39495 (12)	1.09955 (15)	0.0285 (4)	
H23	1.0207	0.4240	1.1376	0.034*	
C24	0.89110 (13)	0.35018 (11)	1.15538 (14)	0.0250 (3)	
H24	0.9191	0.3489	1.2318	0.030*	
C25	0.78982 (12)	0.30711 (11)	1.09960 (12)	0.0198 (3)	
H25	0.7487	0.2766	1.1383	0.024*	

### Atomic displacement parameters (Å^2^)

**Table:**

	*U*^11^	*U*^22^	*U*^33^	*U*^12^	*U*^13^	*U*^23^
F1	0.0323 (5)	0.0394 (6)	0.0246 (5)	0.0208 (5)	−0.0003 (4)	−0.0068 (5)
F2	0.0495 (7)	0.0708 (9)	0.0172 (5)	0.0375 (6)	0.0061 (5)	0.0128 (5)
F3	0.0158 (5)	0.0440 (7)	0.0709 (9)	0.0006 (4)	−0.0062 (5)	−0.0050 (6)
F4	0.0241 (4)	0.0307 (5)	0.0174 (5)	−0.0109 (4)	0.0059 (4)	−0.0066 (4)
F5	0.0312 (5)	0.0347 (5)	0.0181 (5)	0.0039 (4)	0.0130 (4)	−0.0075 (4)
F6	0.0283 (5)	0.0250 (5)	0.0139 (4)	−0.0012 (4)	0.0079 (4)	0.0029 (4)
O1	0.0231 (5)	0.0152 (5)	0.0116 (5)	0.0010 (4)	0.0020 (4)	−0.0035 (4)
O2	0.0150 (5)	0.0235 (5)	0.0129 (5)	0.0064 (4)	0.0045 (4)	−0.0003 (4)
O3	0.0198 (5)	0.0188 (5)	0.0097 (5)	0.0005 (4)	0.0025 (4)	0.0007 (4)
N1	0.0155 (5)	0.0164 (6)	0.0118 (6)	−0.0008 (4)	0.0040 (4)	0.0003 (5)
N2	0.0146 (5)	0.0171 (6)	0.0077 (5)	0.0022 (4)	0.0038 (4)	−0.0003 (4)
C1	0.0152 (6)	0.0168 (7)	0.0136 (7)	−0.0004 (5)	0.0031 (5)	0.0007 (5)
C2	0.0162 (6)	0.0167 (7)	0.0130 (7)	0.0004 (5)	0.0049 (5)	−0.0013 (5)
C3	0.0148 (6)	0.0139 (7)	0.0117 (7)	−0.0034 (5)	0.0043 (5)	−0.0001 (5)
C4	0.0145 (6)	0.0133 (6)	0.0119 (6)	−0.0017 (5)	0.0052 (5)	0.0007 (5)
C5	0.0155 (6)	0.0172 (7)	0.0126 (7)	−0.0006 (5)	0.0030 (5)	0.0026 (5)
C6	0.0156 (6)	0.0181 (7)	0.0215 (8)	0.0023 (5)	0.0062 (6)	0.0019 (6)
C7	0.0204 (7)	0.0160 (7)	0.0204 (8)	0.0000 (6)	0.0111 (6)	−0.0014 (6)
C8	0.0172 (6)	0.0142 (6)	0.0139 (7)	−0.0040 (5)	0.0064 (5)	−0.0021 (5)
C9	0.0144 (6)	0.0127 (6)	0.0129 (7)	−0.0025 (5)	0.0052 (5)	−0.0002 (5)
C10	0.0222 (7)	0.0291 (8)	0.0149 (7)	0.0065 (6)	0.0012 (6)	−0.0028 (6)
C11	0.0207 (7)	0.0223 (8)	0.0156 (7)	−0.0029 (6)	0.0090 (6)	−0.0037 (6)
C12	0.0135 (6)	0.0155 (7)	0.0105 (7)	−0.0001 (5)	0.0027 (5)	−0.0003 (5)
C13	0.0140 (6)	0.0148 (6)	0.0093 (6)	0.0000 (5)	0.0028 (5)	−0.0010 (5)
C14	0.0183 (6)	0.0189 (7)	0.0135 (7)	0.0029 (5)	0.0082 (5)	−0.0010 (5)
C15	0.0205 (7)	0.0251 (8)	0.0199 (8)	0.0012 (6)	0.0112 (6)	0.0025 (6)
C16	0.0173 (7)	0.0222 (8)	0.0201 (8)	−0.0037 (6)	0.0059 (6)	0.0029 (6)
C17	0.0133 (6)	0.0187 (7)	0.0144 (7)	−0.0003 (5)	0.0032 (5)	0.0014 (5)
C18	0.0155 (6)	0.0131 (6)	0.0109 (7)	−0.0017 (5)	0.0044 (5)	−0.0012 (5)
C19	0.0181 (7)	0.0172 (7)	0.0179 (7)	0.0052 (6)	0.0037 (5)	−0.0020 (6)
C20	0.0164 (6)	0.0132 (7)	0.0200 (8)	0.0056 (5)	0.0040 (5)	−0.0013 (6)
C21	0.0261 (8)	0.0201 (8)	0.0214 (8)	0.0027 (6)	0.0080 (6)	−0.0016 (6)
C22	0.0260 (8)	0.0214 (8)	0.0367 (10)	−0.0043 (6)	0.0140 (7)	−0.0027 (7)
C23	0.0211 (7)	0.0214 (8)	0.0376 (10)	−0.0011 (6)	0.0031 (7)	−0.0067 (7)
C24	0.0252 (8)	0.0205 (8)	0.0217 (8)	0.0053 (6)	−0.0017 (6)	−0.0021 (6)
C25	0.0204 (7)	0.0179 (7)	0.0187 (8)	0.0051 (6)	0.0034 (6)	0.0019 (6)

### Geometric parameters (Å, °)

**Table:**

F1—C10	1.3284 (19)	C8—C11	1.500 (2)
F2—C10	1.3289 (19)	C12—C13	1.5500 (19)
F3—C10	1.339 (2)	C12—H12	1.0000
F4—C11	1.3485 (17)	C13—C17	1.5335 (18)
F5—C11	1.3407 (16)	C13—H13	1.0000
F6—C11	1.3443 (18)	C14—C15	1.529 (2)
O1—C12	1.4159 (17)	C14—H14A	0.9900
O1—H1O	0.840 (9)	C14—H14B	0.9900
O2—C18	1.3570 (16)	C15—C16	1.539 (2)
O2—C19	1.4600 (16)	C15—H15A	0.9900
O3—C18	1.2218 (17)	C15—H15B	0.9900
N1—C1	1.3176 (18)	C16—C17	1.532 (2)
N1—C9	1.3615 (17)	C16—H16A	0.9900
N2—C18	1.3492 (17)	C16—H16B	0.9900
N2—C13	1.4733 (17)	C17—H17A	0.9900
N2—C14	1.4736 (16)	C17—H17B	0.9900
C1—C2	1.402 (2)	C19—C20	1.508 (2)
C1—C10	1.508 (2)	C19—H19A	0.9900
C2—C3	1.3760 (19)	C19—H19B	0.9900
C2—H2	0.9500	C20—C21	1.394 (2)
C3—C4	1.4250 (19)	C20—C25	1.392 (2)
C3—C12	1.5100 (19)	C21—C22	1.389 (2)
C4—C5	1.4223 (19)	C21—H21	0.9500
C4—C9	1.4266 (19)	C22—C23	1.386 (3)
C5—C6	1.363 (2)	C22—H22	0.9500
C5—H5	0.9500	C23—C24	1.383 (2)
C6—C7	1.413 (2)	C23—H23	0.9500
C6—H6	0.9500	C24—C25	1.389 (2)
C7—C8	1.367 (2)	C24—H24	0.9500
C7—H7	0.9500	C25—H25	0.9500
C8—C9	1.4288 (19)		
			
C12—O1—H1O	111.6 (13)	N2—C13—H13	108.1
C18—O2—C19	115.08 (11)	C17—C13—H13	108.1
C1—N1—C9	116.48 (12)	C12—C13—H13	108.1
C18—N2—C13	122.22 (11)	N2—C14—C15	109.92 (11)
C18—N2—C14	117.92 (11)	N2—C14—H14A	109.7
C13—N2—C14	116.21 (11)	C15—C14—H14A	109.7
N1—C1—C2	125.58 (13)	N2—C14—H14B	109.7
N1—C1—C10	114.54 (12)	C15—C14—H14B	109.7
C2—C1—C10	119.77 (12)	H14A—C14—H14B	108.2
C3—C2—C1	118.75 (13)	C14—C15—C16	110.48 (11)
C3—C2—H2	120.6	C14—C15—H15A	109.6
C1—C2—H2	120.6	C16—C15—H15A	109.6
C2—C3—C4	118.39 (12)	C14—C15—H15B	109.6
C2—C3—C12	119.48 (12)	C16—C15—H15B	109.6
C4—C3—C12	122.08 (12)	H15A—C15—H15B	108.1
C3—C4—C5	123.78 (13)	C17—C16—C15	110.75 (12)
C3—C4—C9	117.62 (12)	C17—C16—H16A	109.5
C5—C4—C9	118.60 (12)	C15—C16—H16A	109.5
C6—C5—C4	120.76 (13)	C17—C16—H16B	109.5
C6—C5—H5	119.6	C15—C16—H16B	109.5
C4—C5—H5	119.6	H16A—C16—H16B	108.1
C5—C6—C7	120.77 (13)	C16—C17—C13	109.86 (11)
C5—C6—H6	119.6	C16—C17—H17A	109.7
C7—C6—H6	119.6	C13—C17—H17A	109.7
C8—C7—C6	120.40 (13)	C16—C17—H17B	109.7
C8—C7—H7	119.8	C13—C17—H17B	109.7
C6—C7—H7	119.8	H17A—C17—H17B	108.2
C7—C8—C9	120.34 (13)	O3—C18—N2	125.34 (12)
C7—C8—C11	120.33 (13)	O3—C18—O2	122.70 (12)
C9—C8—C11	119.31 (12)	N2—C18—O2	111.96 (11)
N1—C9—C4	123.10 (12)	O2—C19—C20	112.88 (12)
N1—C9—C8	117.80 (12)	O2—C19—H19A	109.0
C4—C9—C8	119.10 (12)	C20—C19—H19A	109.0
F2—C10—F1	107.32 (14)	O2—C19—H19B	109.0
F2—C10—F3	106.66 (14)	C20—C19—H19B	109.0
F1—C10—F3	106.61 (13)	H19A—C19—H19B	107.8
F2—C10—C1	112.90 (13)	C21—C20—C25	119.02 (14)
F1—C10—C1	112.91 (12)	C21—C20—C19	120.30 (14)
F3—C10—C1	110.05 (13)	C25—C20—C19	120.68 (13)
F5—C11—F6	106.16 (11)	C22—C21—C20	120.15 (15)
F5—C11—F4	106.16 (12)	C22—C21—H21	119.9
F6—C11—F4	106.48 (12)	C20—C21—H21	119.9
F5—C11—C8	111.76 (12)	C23—C22—C21	120.35 (15)
F6—C11—C8	113.08 (12)	C23—C22—H22	119.8
F4—C11—C8	112.69 (11)	C21—C22—H22	119.8
O1—C12—C3	107.77 (11)	C24—C23—C22	119.87 (15)
O1—C12—C13	111.73 (11)	C24—C23—H23	120.1
C3—C12—C13	109.35 (11)	C22—C23—H23	120.1
O1—C12—H12	109.3	C23—C24—C25	119.97 (15)
C3—C12—H12	109.3	C23—C24—H24	120.0
C13—C12—H12	109.3	C25—C24—H24	120.0
N2—C13—C17	109.02 (10)	C24—C25—C20	120.64 (14)
N2—C13—C12	110.44 (11)	C24—C25—H25	119.7
C17—C13—C12	113.07 (11)	C20—C25—H25	119.7
			
C9—N1—C1—C2	−0.3 (2)	C9—C8—C11—F4	−63.18 (18)
C9—N1—C1—C10	−176.49 (12)	C2—C3—C12—O1	−24.67 (16)
N1—C1—C2—C3	−2.3 (2)	C4—C3—C12—O1	157.75 (12)
C10—C1—C2—C3	173.71 (13)	C2—C3—C12—C13	96.96 (15)
C1—C2—C3—C4	2.6 (2)	C4—C3—C12—C13	−80.62 (15)
C1—C2—C3—C12	−175.10 (12)	C18—N2—C13—C17	136.12 (13)
C2—C3—C4—C5	178.88 (13)	C14—N2—C13—C17	−21.86 (16)
C12—C3—C4—C5	−3.5 (2)	C18—N2—C13—C12	−99.08 (14)
C2—C3—C4—C9	−0.51 (19)	C14—N2—C13—C12	102.94 (13)
C12—C3—C4—C9	177.10 (12)	O1—C12—C13—N2	−67.05 (13)
C3—C4—C5—C6	−177.76 (13)	C3—C12—C13—N2	173.73 (10)
C9—C4—C5—C6	1.6 (2)	O1—C12—C13—C17	55.41 (15)
C4—C5—C6—C7	−0.7 (2)	C3—C12—C13—C17	−63.81 (14)
C5—C6—C7—C8	−0.4 (2)	C18—N2—C14—C15	−94.29 (15)
C6—C7—C8—C9	0.6 (2)	C13—N2—C14—C15	64.67 (15)
C6—C7—C8—C11	−178.42 (13)	N2—C14—C15—C16	−37.26 (16)
C1—N1—C9—C4	2.5 (2)	C14—C15—C16—C17	−23.02 (16)
C1—N1—C9—C8	−177.60 (12)	C15—C16—C17—C13	65.54 (15)
C3—C4—C9—N1	−2.2 (2)	N2—C13—C17—C16	−41.29 (15)
C5—C4—C9—N1	178.41 (12)	C12—C13—C17—C16	−164.53 (12)
C3—C4—C9—C8	177.97 (12)	C13—N2—C18—O3	−165.29 (13)
C5—C4—C9—C8	−1.45 (19)	C14—N2—C18—O3	−7.7 (2)
C7—C8—C9—N1	−179.50 (13)	C13—N2—C18—O2	15.20 (18)
C11—C8—C9—N1	−0.48 (19)	C14—N2—C18—O2	172.82 (11)
C7—C8—C9—C4	0.4 (2)	C19—O2—C18—O3	7.38 (19)
C11—C8—C9—C4	179.39 (12)	C19—O2—C18—N2	−173.10 (11)
N1—C1—C10—F2	−38.11 (19)	C18—O2—C19—C20	−82.15 (15)
C2—C1—C10—F2	145.43 (15)	O2—C19—C20—C21	−75.11 (16)
N1—C1—C10—F1	−160.08 (13)	O2—C19—C20—C25	106.09 (15)
C2—C1—C10—F1	23.5 (2)	C25—C20—C21—C22	0.9 (2)
N1—C1—C10—F3	80.93 (17)	C19—C20—C21—C22	−177.93 (14)
C2—C1—C10—F3	−95.52 (16)	C20—C21—C22—C23	−0.2 (2)
C7—C8—C11—F5	−3.62 (19)	C21—C22—C23—C24	−0.5 (2)
C9—C8—C11—F5	177.36 (12)	C22—C23—C24—C25	0.5 (2)
C7—C8—C11—F6	−123.34 (14)	C23—C24—C25—C20	0.2 (2)
C9—C8—C11—F6	57.64 (16)	C21—C20—C25—C24	−0.9 (2)
C7—C8—C11—F4	115.84 (14)	C19—C20—C25—C24	177.91 (13)

### Hydrogen-bond geometry (Å, °)

**Table:**

*D*—H···*A*	*D*—H	H···*A*	*D*···*A*	*D*—H···*A*
O1—H1o···O3^i^	0.84 (1)	1.90 (1)	2.7294 (14)	172.(2)

Symmetry codes: (i) −*x*+1, −*y*+1, −*z*+2.
